# Natural Killer Cells in Viral Infection: Special Issue Editorial

**DOI:** 10.3390/v17030391

**Published:** 2025-03-10

**Authors:** Min Fang

**Affiliations:** School of Life Sciences, Henan University, Kaifeng 475001, China; fangmin@henu.edu.cn

NK cells are a critical cell population in innate immunity, playing an important role in antiviral immunity. NK cells function by directly killing viral-infected cells and by producing various cytokines to regulate immune responses. NK cell activation is tightly regulated by the engagement of its inhibitory and activating receptors. Our knowledge of NK cell recognition and regulatory mechanisms has been gained partly from viral infections. However, further research is still needed on how NK cells recognize different viral-infected cells, how NK cells interact with other immune cells to form a complex regulatory network, and the mechanisms regulating NK cell memory.

The first paper (https://www.mdpi.com/1999-4915/16/7/1167 (accessed on 20 July 2024)) in this Special Issue reveals the need of co-activating receptors for effective ADCC (antibody-dependent cellular cytotoxicity) mediated by NK cells to kill HIV-infected CD4 cells. Despite the success of antiretroviral therapy, HIV-1-infected individuals still harbor latent viruses. Eliminating virus-infected cells will reduce the viral reservoir. In addition to neutralizing antibodies, non-neutralizing antibodies (nnAbs) are abundant in the plasma of people living with HIV. Using the small CD4-mimetic compound (CD4mc) to expose HIV Env epitopes recognized by nnAbs results in potent NK cell ADCC activities [1]. Co-activating receptors NTB-A and 2B4 sustain the CD16 (FcγRIIIa)-mediated ADCC. Even the ligands are partially downregulated by HIV, NTB-A and 2B4 significantly contribute to CD4mc sensitization of HIV-1-infected cells to ADCC. This study highlighted the importance of co-activating receptors in NK cell ADCC to eliminate HIV-infected cells, and a potential strategy to reduce viral reservoirs.

The second paper (https://www.mdpi.com/1999-4915/16/5/741 (accessed on 8 May 2024)) focused on the crosstalk between NK cells and monocytes during hepatitis B virus (HBV) infection. Chronic HBV infection is one of the main causes of liver cirrhosis worldwide. NK cells play an important role in direct cytotoxicity and secretion of antiviral cytokines during HBV infection. Using an in vitro cell infection model, the authors demonstrated that the cytokine (IFN-γ and TNF-α) secretion of NK cells to HBV-infected cells is dependent on their interaction with monocytes. The importance of crosstalk between monocytes and NK cells has been emphasized in HCV infection [2,3]. This study indicated that the NK cell-monocyte interactions might play an important role in NK cell anti-viral function during HBV infection. Nevertheless, the mechanisms underlying the crosstalk between NK cells and monocytes still require further investigation.

The third paper (https://www.mdpi.com/1999-4915/16/5/737 (accessed on 7 May 2024)) identified that Gal-3-ITGB1 signaling mediates IL-10 production of cNK cells in HBV-transgenic mice. Furthermore, LGALS3 and ITGB1 expression correlated with poor progression and survival of hepatocellular carcinoma patients. The hepatic NK cells harbor two distinct subsets—CD49a^−^CD49b^+^ conventional NK (cNK) cells and D49a^+^CD49b^−^ liver-resident NK (LrNK) cells [4,5]. This study elucidated a molecular mechanism for regulating hepatic NK cell subset functions during chronic HBV infection, revealing that interrupting the Gal-3-ITGB1 interaction may help control HBV-related liver disease.

The fourth paper (https://www.mdpi.com/1999-4915/16/11/1746 (accessed on 7 November 2024)) summarized the current knowledge about the generation of memory NK cells and their clinical application in viral infection and cancer. Antigen-specific memory NK cell responses were first demonstrated in contact hypersensitivity to haptens in a mouse model. Those hapten-specific memory NK cells resided in the liver and were Thy1^+^Ly49C/I^+^ [6]. The adaptive immune features of NK cells were clearly demonstrated in MCMV and HCMV infections, with specific NK cell activating receptors involved in recognizing virus-infected target cells [7,8]. In addition to viral infection, bacterial infection can also generate antigen-specific memory-like features in NK cells [9]. Furthermore, cytokine-induced memory-like NK cells have been combined with CAR therapy. Incorporating memory NK cells into immunotherapy against viral infection and malignancy is an attractive strategy.

The fifth paper (https://www.mdpi.com/1999-4915/16/10/1584 (accessed on 9 October 2024)) is a comprehensive review of the role of NK cells in HIV infection. NK cells are potent anti-HIV-1 immune cells [10], and understanding their function and regulation mechanisms can allow us to better harness the antiviral and immunoregulatory function of NK cells to intervene in the progression of HIV infection. The review includes NK cell-mediated immunity to HIV-1 infection and viral evasion mechanisms, and consequences of HIV-1 infection on NK cells. The authors also emphasized the critical link between cellular metabolism and NK cell immune function and indicated that identifying novel metabolic pathways that alleviate NK cell impairment might serve as targets for therapies that reconstitute and enhance NK cell functions in people living with HIV.

This Special Issue provides a fascinating snapshot of NK cell functions during viral infection, especially HIV and HBV infection. The research involves the molecular mechanisms regulating NK cell functions, including ADCC and cytokine production, as well as the interactions between NK cells and other immune cells. These two review articles summarize the research progress on memory NK cells and NK cells in HIV infection, two very important research areas. Understanding the recognition and regulatory mechanisms of NK cells will help to fully leverage their powerful antiviral functions.
